# Is the Occurrence or Reversal of Nonalcoholic Fatty Liver Disease Associated with Long-Term Helicobacter pylori Infection among Chinese Adults? A Cohort Study

**DOI:** 10.1155/2021/6696473

**Published:** 2021-11-24

**Authors:** Xia-Xia Zhao, Rui-Ling Wang, Ming-Hao Liu, Xiao-jun Huang

**Affiliations:** ^1^Department of Gastroenterology, Lanzhou University Second Hospital, No. 82 Cuiying Men, Cheng Guan District, Lanzhou, 730030 Gansu Province, China; ^2^The PLA Rocket Force Characteristic Medical Center, Digestive Internal Medicine, Beijing, China

## Abstract

**Background:**

Previous studies have suggested a link between Helicobacter pylori (H. pylori) infection and nonalcoholic fatty liver disease (NAFLD), yet long-term follow-up studies to elucidate this association are lacking. We aimed to identify the relationship between NAFLD and H. pylori in these people.

**Methods:**

A total of 2,934 adults between June 2013 and October 2017 were collected; among them, 675 people met the requirements. People were assessed for H. pylori infection diagnosis as detected by the carbon-13 urea breath test; they were also assessed for NAFLD diagnosis by ultrasound.

**Results:**

H. pylori infection was present in 206 patients (30.5%), and 469 (69.5%) participants were classified as controls. Participants with H. pylori infection had a higher rate of incident NAFLD than those who were uninfected (37/206; 18% versus 73/469; 15.6%) (*p* < 0.001). Compared with the control group, the recovery rate of NAFLD in the H. pylori+ve group was low (6/206, 2.9% versus 33/469, 7.0%) (*p* < 0.001). Besides, the incidence of uric acid, postprandial blood glucose, TG, LDL-C, HDL-C, and fasting plasma glucose was significantly different between the two groups (*p* < 0.001), but no difference was found in alanine aminotransferase (ALT), liver-total protein, urea nitrogen, and cholesterol (*p* > 0.05).

**Conclusion:**

H. pylori infection was a risk factor for NAFLD and affected the occurrence or reversal of NAFLD, indicating that H. pylori infection eradication might play a role in reducing the risk of NAFLD.

## 1. Introduction

Helicobacter pylori (H. pylori) is a gram-negative bacterium. There are approximately 4.4 billion individuals diagnosed with H. pylori+ve worldwide; the infection exhibits a high estimated global prevalence; its current average prevalence is assessed to be about 58% (varying from 39.9% to 91.7%) [1, 2]. In China, about half of the population are infected with H. pylori [3–5]. H. pylori infection usually happens in the early phase of life and persists throughout the host's life if untreated [4]. Clinical manifestations of H. pylori infection include peptic ulcer disease, gastric mucosa-associated lymphoid tissue, noncardiac gastric adenocarcinoma, and even stomach cancer [5]. Notably, H. pylori infection not only affects the stomach but is linked to several extragastric diseases.

There is evidence supporting the association of H. pylori infection with NAFLD. Similarly to the high prevalence of nonalcoholic fatty liver disease (NAFLD) [6, 7], hepatosteatosis is defined as the fat accumulation of at least 5% of liver weight. Without excess alcohol intake, this condition is called nonalcoholic fatty liver disease (NAFLD), with a prevalence ranging from 17% to 33% in European countries such as the UK and about 4% to 25% in China [8, 9], being even higher in specific populations, e.g., patients with type 2 diabetes mellitus (T2DM) or obesity [7]. The natural history of NAFLD ranges from simple steatosis (nonalcoholic fatty liver) to nonalcoholic steatohepatitis (NASH) with or without fibrosis, which may progress to cirrhosis and hepatocellular carcinoma [6]. Studies have shown that H. pylori infection has been associated with increased levels of proinflammatory cytokines and IR. Evidence suggests that H. pylori are involved in the regulation of gastric hormones leptin and ghrelin that affect insulin sensitivity and adiposity [10]. Another study from India has revealed a higher prevalence of H. pylori infection in diabetes than controls [11]. The systemic impact of H. pylori infection has received increasing attention [10, 12]. Recent data suggest a possible role of H. pylori in the pathogenesis of NAFLD [13]. Other studies have reported that H. pylori infection may be an important risk factor for NAFLD [14–17]; additionally, our previous study indicated similar results [18]. However, previous studies have not consistently observed the effects of H. pylori on NAFLD in the long term in China. Long-time cohort studies are needed to elucidate the association between H. pylori infection and NAFLD. If proven to be a significant risk factor for NAFLD, H. pylori infection has therapeutic potential, as it can be eradicated in most patients.

The incidences of NAFLD are rapidly increasing, leading to increased clinical and economic burdens [19]. Identifying risk factors with potential therapeutic implications is important in managing NAFLD and decreasing these burdens. In this study, we collected the five-year physical examination indexes of participants who visited our hospital to explore H. pylori infection's effects on NAFLD.

## 2. Materials and Methods

### 2.1. Statement of Ethics

This is a retrospective cohort study. Patients consented to the charts' review, and their information was not disclosed outside our team. Our research was in line with the Helsinki declaration's ethical guidelines (as revised in Brazil 2013), as reflected in the prior approval of our agency's human research council.

### 2.2. Inclusion and Exclusion Criteria

Inclusion criteria were complete, having not used proton pump inhibitors (PPIs), histamine type 2 receptor antagonists (H2A), antibiotics, bismuth, or sucralfate for up to one month before the ^13^C-UBT (carbon-13 urea breath test).

Individuals who had received complete eradication of H. pylori, antibiotics, chronic liver disease, or cirrhosis were excluded. Other exclusion criteria include a self-reported history of malignancy; positive serologic markers for hepatitis B virus; the history of alcohol overconsumption (<210 g per week in men and <140 g per week in women during the past 12 months); and no long-term history of taking steatogenic medications, i.e., amiodarone, methotrexate, tamoxifen, and glucocorticoids. Exclusion of diseases can lead to the fatty liver such as genotype 3 HCV infection, Wilson's disease, autoimmune hepatitis, total parenteral nutrition, hypo-*β*-lipoproteinemia, congenital lipodystrophy, and celiac disease. Participants with missing data on important covariates were excluded.

### 2.3. Study Population

This study included healthy adults, aged 20 years or older, who participated in a comprehensive health screening exam at the General Hospital of PLA Rocket Force from June 2013 to October 2017. Since our objective was to evaluate the longitudinal association between H. pylori infection and NAFLD, we included subjects who underwent screening exams every year, including abdominal ultrasonography (US), to assess the fatty liver status and establish the baseline of H. pylori infection status. About 2,934 people coming to our hospital for physical examination every year were asked to take blood tests, abdominal ultrasonography (US), and complete ^13^C-UBT to diagnose active H. pylori infection. Biochemical investigation, including ALT, total protein, albumin, cholesterol, TG, HDL-C, LDL-C, urea nitrogen, uric acid, fasting plasma glucose, and postprandial blood sugar, was performed in all individuals. After a 12 h overnight fast, 15 mL of blood was collected from the antecubital vein. It was analyzed within 4 hours after collection for biochemistry tests. After an overnight fast, ^13^C-UBT was performed using the Proto Pylori kit (Isodiagnostika Canada), containing 75 mg of ^13^C-UBT and additives. Two breath samples were collected within a 30-minute interval. Patient samples were analyzed by gas chromatography. The results were expressed as delta over baseline (DOB). NAFLD was examined using a Philips HD 11 XE multifunction color Doppler diagnostic instrument, according to the new standard criteria for NAFLD by China, 2018 [19]. The same ultrasound physician performed all ultrasound measurements throughout the study. The diagnostic criteria are as follows: ALT > 40 U/L, total protein > 5.69 mmol/L, cholesterol > 5.69 mmol/L, TG > 1.7 mmol/L, HDL − C < 1.7 mmol/L, LDL − C > 3.64 mmol/L, urea nitrogen > 8.05 mmol/L, creatinine > 115 mmol/L, uric acid > 416 mol/L, fasting plasma glucose > 6.1 mmol/L, and postprandial plasma glucose > 7.8 mmol/L. The results of ^13^C-UBT are expressed as DOB values; DOB ≥ 4 was H. pylori+ve, and DOB < 4 was H. pylori-ve. NAFLD and Mets were diagnosed according to the latest guidelines [19, 20]. Since the same people have physical examinations in our hospital every year, we have the opportunity to collect and identify their data over a long period of time.

### 2.4. Statistical Analysis

SPSS software version 22.0 (SPSS Inc., Chicago, IL) was used for statistical analysis. The Student *t*-test and chi-square test were used to analyze the distribution of continuous variables and classified variables. The data are represented as the mean ± standard deviation, and *p* < 0.05 was considered statistically significant.

## 3. Results

### 3.1. Data Collection

**Figure 1:**
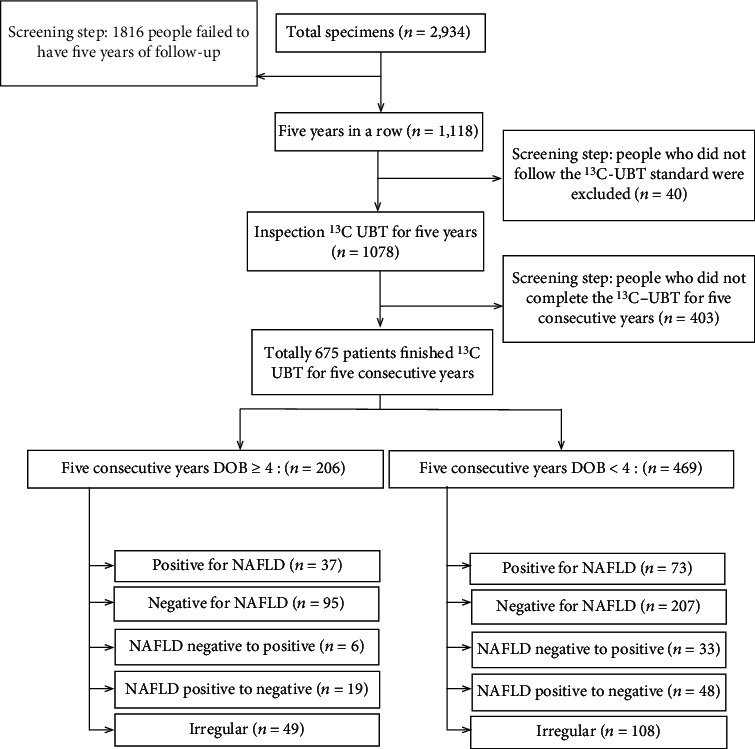
Diagram of patients included in the study. DOB: delta over baseline; NAFLD: nonalcoholic fatty liver disease.

**Figure 2:**
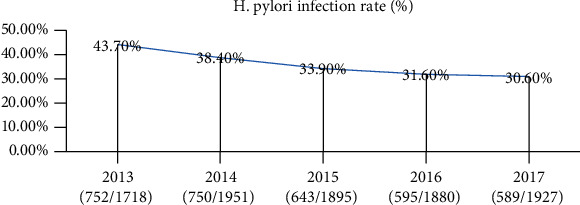
The trend of H. pylori infection rate in five years.

A total of 2,934 participants were collected. People who did not meet the inclusion criteria were removed (*n* = 1,816), the remaining 1,118 people were included, and among them, additional 40 people were excluded due to the ^13^C-UBT standard which is not followed; eventually, 1,078 people were left and had completed the ^13^C-UBT for 5-year follow-up. After excluding the participants (*n* = 403) who did not complete ^13^C-UBT for 5 years, in total, 675 (males/females: 506/169; aged 54.7 ± 8.6 years) subjects who met these criteria were selected. The further screening revealed 206 individuals with DOB ≥ 4 and 469 people with DOB < 4, and the specific groups are shown in Figure 1. Characteristics of all individuals are presented in Table 1, the continuous prevalence of H. pylori positivity was found to be 30.5% for 5 years (206/675), and the infection rate of H. pylori gradually decreased from 2013 to 2017 (Figure 2); NAFLD was found to be 31.9% (110/675).

### 3.2. General Characteristics of H. pylori Infection

The characteristics classified being H. pylori+ve or H. pylori-ve are shown in Table 2. In this study, participants with NAFLD had a higher rate of H. pylori infection than those without NAFLD (27/206; 18% versus73/469; 15.6%). When H. pylori+ve and H. pylori-ve individuals were compared, postprandial blood sugar (17/206; 8.3% versus 30/469; 6.4%), HDL-C (14/206; 6 .8% versus 16/469; 3 .4%), and uric acid (14/206; 6.8% versus 20/469; 4 .3%) were found to be significantly higher in H. pylori+ve patients. Additionally, we could clearly find that the prevalence of LDL-C in the group with and without H. pylori was 106 patients (51.5%) and 235 patients (50.1%), in which this difference was significant, fasting plasma glucose, in this cohort, also showed a statistically significant difference (*p* < 0.001). According to the TG levels, there was a significant difference in the two groups (20/206; 9.7% versus 39/469; 8.3%), while no difference was found between the two groups in terms of ALT (0.518), total protein (0.087), urea nitrogen (0.518), and cholesterol (0.497). Also, both groups predominantly consisted of males, while the ratio of males in the H. pylori+ve group (152/206; 73.7%) was lower than that in the control group (354/469; 75.4%) (*p* = 0 026).

### 3.3. Annual NAFLD Based on H. pylori Status

**Figure 3:**
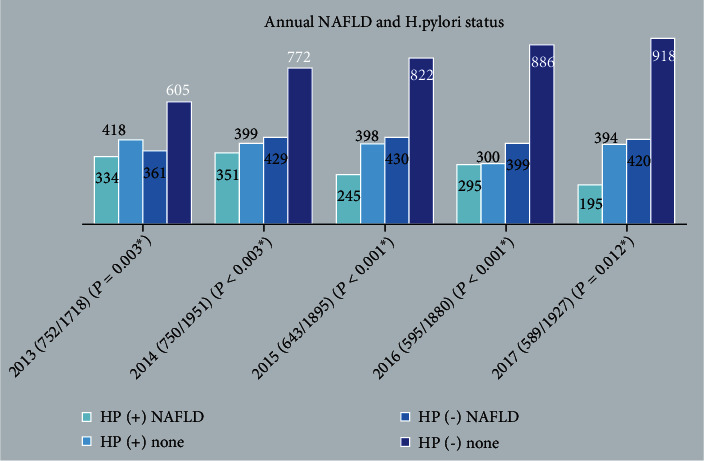
Correlation between H. pylori and NAFLD every year; ∗ means *p* is less than 0.05.

In separate annual observational studies, there were about 2,934 individuals; among them, those who were not tested for ^13^C-UBT were excluded. Finally, the status of H. pylori infection in the population from June 2013 to October 2017 was shown as follows: 752/1,718 (43.7%), 750/1,951 (38.4%), 643/1,895 (33.9%), 589/1,867 (31.5%), and 589/1,927 (30.6%) (Figure 3). The prevalence of NAFLD in the group with and without H. pylori has statistically significant differences every year (*p* < 0.01) (Figure 3).

### 3.4. The Relationship between the Occurrence and Recovery of NAFLD and H. pylori

Individuals diagnosed as H. pylori+ve were divided into 5 groups in our five-year follow-up according to NAFLD status as follows: people with NAFLD (37/206, 18.0%), participants without NAFLD (95/206, 46.1%), patients with NAFLD recovering to without (19/206, 9.2%), people from without NAFLD to with (6/206, 2.9%), and irregular changes between the onset and recovery of NAFLD within 5 years (49/206, 23.8%). In the same way, patients diagnosed as H. pylori-ve were also divided into five groups as below: patients with NAFLD (73/469, 15.6%), people without NAFLD (207/469, 44.1%), patients with NAFLD recovering to without (48/469, 10.2%), people from without NAFLD to with (33/469, 7.0%), and the morbidity and rehabilitation of NAFLD were irregular (108/469, 20.7%). Statistical results are shown in Table 3. We found that the recovery rate of NAFLD in the H. pylori+ve group was lower than that in the control group (6/206; 2.9% versus 33/469; 7.0%) (*p* < 0.001), indicating that H. pylori infection significantly decreased the cure of NAFLD, while there were no statistical differences in the recovery of NAFLD and H. pylori infection between the participants within 5 years (*p* = 0.36).

## 4. Discussion

In this cohort study of the association between H. pylori infection and the risk of incident NAFLD, we found that the infection rate of H. pylori was observed to gradually decrease from 2013 to 2017 in the patient population (Figure 2). The continuous infection rate of H. pylori for 5 years was 30.5% (Table 1), which is lower than that for the general Chinese population (39%-48%), possibly due to living in crowded conditions and low socioeconomic status of the participants [21, 22]. In terms of gender, women have lower rates of H. pylori infection than men, Chen et al. observed that female is an independent protective factor for H. pylori infection [23]; similar results were also observed in our study (Table 2), which could be due to the gender-related hormonal differences. H. pylori infection can cause Mets [24, 25]. A cohort study on 17,028 participants showed that lipid metabolism markers, such as LDL-C, HDL-C, and TG, were significantly associated with H. pylori [16, 26], which is consistent with our finding that elevated LDL-C and TG but decreased HDL-C were observed in H. pylori+ve individuals. This may be because H. pylori infection can enhance oxidative stress, subsequently affecting the insulin signaling pathway through multiple ways, such as inhibiting the activation of fatty acyl inositol 3 kinase p85 subunits, preventing the transport of glucose transporter-4 from the vesicle to the plasma membrane, and downregulating the expression of glucose transporter protein-4, thereby leading to IR and eventually causing dyslipidemia and glucose metabolism disorders [27, 28]. It is worth noting that this study showed that TG, uric acid levels, and diabetes are significantly associated with H. pylori infection in 205 patients [22], consistent with our findings. Still, urea nitrogen, cholesterol, ALT, and total protein metabolism abnormalities are not associated with H. pylori infection (*p* > 0.05) (Table 2). More studies are needed to confirm these results further.

NAFLD is an acquired metabolism-related liver injury, related to an increased risk of chronic kidney disease, type 2 diabetes, and cardiovascular disease [5]. There has been an intensive debate on the associations and causalities between H. pylori and NAFLD in recent years. A retrospective study on 3,663 patients in South Korea demonstrated that H. pylori infection was a risk factor for NAFLD [29]. A similar conclusion was reported in another study based on biopsy analysis from patients with H. pylori infection [30]. This may be caused by H. pylori-induced interleukin-1 (IL-1), IL-6, tumor necrosis factor, interferon, and c-reactive protein, which can cause hepatocyte damage; another possible reason is that H. pylori infection can increase intestinal permeability and facilitate the passage of bacterial toxins through the portal vein to the liver [31, 32]. It is worth noting that some inconsistent findings were also reported. Studies in South Korea and Japan showed that body mass index, smoking, and c-reactive protein concentration were risk factors for NAFLD, but H. pylori infection did not increase NAFLD risk [33, 34]. Our study supported the first point and showed that NAFLD had a higher rate in H. pylori-positive patients (*p* < 0.05) (Tables 2 and 3). NAFLD is more difficult to recover to normal under H. pylori infection situation (*p* < 0.001) (Table 3). These results may vary depending on participants' lifestyles, diet, and physical activity. Additionally, the host's health, the intensity of inflammatory response, autoimmune response, and antioxidant protection are all related to H. pylori infection [15, 35]. Moreover, different H. pylori detection technologies and specimen locations may lead to significant differences in test results [36, 37]. However, there was no significant statistical difference in the incidence of new NAFLD within five years (*p* = 0.36) (Table 3), which may be due to the insufficient follow-up time or small sample size.

NAFLD, which is currently renamed to metabolic- (dysfunction) associated fatty liver disease (MAFLD) [38], is closely associated with metabolic syndrome (Mets). There is a big overlap between NAFLD and Mets [39, 40]. NAFLD is mutually and bidirectionally linked with Mets, and it is both the cause and the consequence of Mets [36]. These conditions share similar pathophysiological mechanisms and risk factors [41–43]. More specifically, IR and inflammation link these conditions to each other. Along with IR, some other pathophysiological mechanisms, e.g., disordered lipid metabolism, increased oxidative stress, and inflammation, link NAFLD to Mets [44]. Among the metabolic variables, lipid metabolism markers, such as LDL-C, HDL-C, and the combination of LDL-C, HDL-C, and triglycerides, were significant mediators of the association between H. pylori infection and NAFLD [23]. It is worth noting that H. pylori infection may increase oxidative stress and may be linked to chronic inflammation, IR, and disturbance of lipid metabolism [10, 22, 27, 45]. Besides, the Mets risks that H. pylori are known to cause are most of all a consequence of insulin resistance and its comorbidities, which, in turn, are closely associated with NAFLD. Thereby, eradication of H. pylori may be an important step to prevent Mets and reduce NAFLD. The exact mechanism linking H. pylori infection and NAFLD to Mets needs to be further studied in more detail. However, the limitations of the present study must be noted: this is a retrospective analysis of our hospital database without further adjustment for other factors such as age, economic status, and living environment, which may affect this study' results. Despite the limitations, our study has some advantages. To our knowledge, our study is the first to follow up NAFLD for five consecutive years in China, further confirming the correlationships between H. pylori infection and NAFLD.

In conclusion, H. pylori are the highway for Mets and NAFLD, resulting in a serious public health problem worldwide. Therefore, patients infected with H. pylori need to be highly vigilant and timely to identify NAFLD. From a long-range point of view, it is of utmost importance to eradicate H. pylori infection in subjects who may be at increased risk for future Mets or NAFLD, which may help prevent Mets and NAFLD and therefore provides novel insights for effective prevention and treatment of NAFLD.

## Figures and Tables

**Table 1:** Characteristics of all participants based on H. pylori infection.

	Mean ± SD
Age (years)	54.7 ± 8.6 (years)
ALT (U/L)	19.8 ± 11.9 (U/L)
Total protein (mmol/L)	72.3 ± 4.0 (mmol/L)
Cholesterol (mmol/L)	4.8 ± 0.9 (mmol/L)
TG (mmol/L)	1.5 ± 0.1 (mmol/L)
HDL-C (mmol/L)	1.3 ± 0.3 (mmol/L)
LDL-C (mmol/L)	3.2 ± 0.8 (mmol/L)
Urea nitrogen (mmol/L)	5.4 ± 1.4 (mmol/L)
Uric acid (mmol/L)	336 ± 79.1 (mmol/L)
Postprandial blood sugar (mmol/L)	6.9 ± 2.2 (mmol/L)
Fasting plasma glucose (mmol/L)	5.2 ± 1.0 (mmol/L)
H. pylori+ve	206 (30.5%) (mmol/L)
NAFLD	110 (16.3%)

**Table 2:** Characteristics of categorical variables broken down by H. pylori status (统计占比).

	Total	H. pylori (+)	H. pylori (−)	*p* value
	Number	Number	Number	
Gender (male/female)	506/169	152/54	354/115	0.026^∗^
Postprandial blood glucose (mmol/L)	47 (7.0%)	17 (8.3%)	30 (6.4%)	<0.001^∗^
ALT (U/L)	2 (0.3%)	1 (0.5%)	1 (0.2%)	0.518
Total protein (mmol/L)	671 (99.4%)	203 (98.5%)	468 (99.8)	0.087
Cholesterol (mmol/L)	152 (2%)	4 (19.4%)	11 (2.3%)	0.497
TG (mmol/L)	59 (8.7%)	20 (9.7%)	39 (8.3%)	<0.001^∗^
HDL-C (mmol/L)	30 (4.4%)	14 (6.8%)	16 (3.4%)	<0.001^∗^
LDL-C (mmol/L)	341 (50.5%)	106 (51.5%)	235 (50.1%)	<0.001^∗^
Urea nitrogen (mmol/L)	2 (0.3%)	1 (0.5%)	1 (0.2%)	0.518
Uric acid (mmol/L)	34 (5.0%)	14 (6.8%)	20 (4.3%)	<0.001^∗^
Fasting plasma glucose (mmol/L)	20 (3.0%)	7 (3.4%)	13 (2 .8%)	<0.001^∗^
NAFLD	110 (16.3%)	37 (18%)	73 (15.6%)	<0.001^∗^

∗ represents that the index was statistically significant among the participants with H. pylori+ve.

**Table 3:** The relationship between the occurrence and recovery of NAFLD and H. pylori.

Variable	H. pylori (+)	H. pylori (-)	*p* value
Positive to negative	19 (9.2%)	48 (10.2%)	
Positive	37 (18.0%)	73 (15.6%)	0.36
Negative to positive	6 (2.9%)	33 (7.0%)	
Negative	95 (46.1%)	207 (44.1%)	<0.001^∗^

∗ means *p* is less than 0.05.

## Data Availability

The data used to support the findings of this study are available from the corresponding author upon request.
